# Improved hybrid *de novo* genome assembly of domesticated apple (*Malus x domestica*)

**DOI:** 10.1186/s13742-016-0139-0

**Published:** 2016-08-08

**Authors:** Xuewei Li, Ling Kui, Jing Zhang, Yinpeng Xie, Liping Wang, Yan Yan, Na Wang, Jidi Xu, Cuiying Li, Wen Wang, Steve van Nocker, Yang Dong, Fengwang Ma, Qingmei Guan

**Affiliations:** 1 State Key Laboratory of Crop Stress Biology for Arid Areas, College of Horticulture, Northwest A&F University, Yangling, 712100 China; 2 State Key Laboratory of Genetic Resources and Evolution, Kunming Institute of Zoology, Chinese Academy of Sciences, Kunming, 650223 China; 3 Agri-biotech-lab Company, Kunming, 650220 China; 4 Department of Horticulture, Michigan State University, East Lansing, MI 48824 USA; 5 College of Biological big data, Yunnan Agriculture University, Kunming, 650504 China; 6 Faculty of Life Science and Technology, Kunming University of Science and Technology, Kunming, 650500 China

**Keywords:** *Malus* x *domestica*, Apple, Illumina sequencing, PacBio sequencing

## Abstract

**Background:**

Domesticated apple (*Malus* × *domestica* Borkh) is a popular temperate fruit with high nutrient levels and diverse flavors. In 2012, global apple production accounted for at least one tenth of all harvested fruits. A high-quality apple genome assembly is crucial for the selection and breeding of new cultivars. Currently, a single reference genome is available for apple, assembled from 16.9 × genome coverage short reads via Sanger and 454 sequencing technologies. Although a useful resource, this assembly covers only ~89 % of the non-repetitive portion of the genome, and has a relatively short (16.7 kb) contig N50 length. These downsides make it difficult to apply this reference in transcriptive or whole-genome re-sequencing analyses.

**Findings:**

Here we present an improved hybrid *de novo* genomic assembly of apple (Golden Delicious), which was obtained from 76 Gb (~102 × genome coverage) Illumina HiSeq data and 21.7 Gb (~29 × genome coverage) PacBio data. The final draft genome is approximately 632.4 Mb, representing ~ 90 % of the estimated genome. The contig N50 size is 111,619 bp, representing a 7 fold improvement. Further annotation analyses predicted 53,922 protein-coding genes and 2,765 non-coding RNA genes.

**Conclusions:**

The new apple genome assembly will serve as a valuable resource for investigating complex apple traits at the genomic level. It is not only suitable for genome editing and gene cloning, but also for RNA-seq and whole-genome re-sequencing studies.

**Electronic supplementary material:**

The online version of this article (doi:10.1186/s13742-016-0139-0) contains supplementary material, which is available to authorized users.

## Data description

### Whole-genome shotgun sequencing of ‘Golden Delicious’ apple on the Illumina platform

Genomic DNA was extracted from leaf tissues of a single ‘Golden Delicious’ apple tree with the GenElute™ Plant Genomic DNA Miniprep Kit (Sigma-Aldrich; St. Louis, USA). Paired-end libraries with insert sizes ranging from 350–500 bp were constructed with Next UltraTM DNA Library Prep Kit for Illumina (NEB; USA) according to the manufacturer’s instructions. These libraries were sequenced on an Illumina HiSeq 4000 platform (Illumina; CA, USA) using the PE-150 module [1], and yielded about 86 Gb of raw data. These data were then subjected to filtering to remove: (1) reads in which more than 5 % of bases were N or poly-A; (2) reads in which more than 30 bases were of low quality; (3) reads with adapter contamination; (4) reads shorter than 30 bp; and (5) PCR duplicates. These steps yielded a clean sequence of ~76 GB, representing about 102 × genome coverage (Additional file 1: Table S1). *De novo* assembly was performed with with SOAPec_v2.01 [2] using default parameters.

## Single-molecule long read sequencing of ‘Golden Delicious’ apple on the PacBio platform

Single-molecule long reads from the PacBio RS II platform (Pacific Biosciences, USA) were used to assist the subsequent *de novo* genome assembly [3]. In brief, 15 μg of sheared DNA was used to construct five SMRT Bell libraries with an insert size of 17 kb. The libraries were then sequenced in 20 single-molecule real-time DNA sequencing cells using the P6 polymerase/C4 chemistry combination, and a data collection time of 240 min per cell. The sequencing produced about 21.7 Gb data, consisting of 2,759,937 reads with an average read length of 7,863 bp (Additional file 1: Figure S1). The polymerase read N50 length after single passing was around 16.6 kb, and the polymerase read quality was greater than 82.4 % (Additional file 1: Table S1).

## Estimation of the ‘Golden Delicious’ apple genome size

Quality-filtered reads from the Illumina platform were subjected to 23-mer frequency distribution analysis with Jellyfish [4]. Analysis parameters were set at -k 23, and the final result was plotted as a frequency graph (Additional file 1: Figure S2). Two distinctive modes were observed from the distribution curve: the higher peak at a depth of 88 reflected the high heterozygosity of the apple genome; the lower peak provided a peak depth of 179 for the estimation of its genome size. Based on the total number of *k*-mers (125,428,662,216), the apple genome size was calculated to be approximately 701 Mb, using the following formula: genome size = *k*-mer_Number/Peak_Depth.

## Hybrid *de novo* genome assembly

A hybrid genome assembly pipeline was used to overcome challenges posed by heterozygous apple genome (Additional file 1: Figure S3). An Illumina-based *de novo* genome assembly was first generated using Platanus [2], yielding a total length of 1.05 Gb, with a contig N50 length of 534 bp. Then, all PacBio RS reads were used in the hybrid assembly process via the DBG2OLC [5] pipeline with the following parameters: LD10, MinLen 200, KmerCovTh 2, MinOverlap 10, AdaptiveTh 0.001, and RemoveChimera 1. This led to a preliminary apple genome assembly of 632.4 Mb with a contig N50 size of 111,619 bp, representing ~90 % of the estimated apple genome (701 Mb). The contig N50 size represents a ~6.9 fold improvement in length from the previously reported 16.1 kb [6]. These improvements were made possible by introducing the long-read sequencing strategy (Additional file 1: Figure S4), which increased the sequencing precision of repeats.

## Evaluation of the completeness of the ‘Golden Delicious’ apple genome assembly

CEGMA was used to evaluate the quality of the final assembly with a set of 248 ultra-conserved core eukaryotic genes [7]. Comparison analysis showed that 231 of 248 genes could be fully annotated (93.15 % completeness, see Table 1), and 243 of 248 genes met the criteria for partial annotation (97.98 % completeness). Using the same evaluation parameters, the completeness of the ‘Golden Delicious’ apple genome assembly v1.0 by Velasco et al. [6] was also evaluated, and a completeness of 88.71 % was obtained (220 of 248 genes could be fully annotated, see Additional file 1: Table S3). This benchmark further demonstrates the improved quality of the genome assembly reported herein.

**Table 1:** Statistics of the completeness of the hybrid *de novo* assembly genome of ‘Golden Delicious’ based on 248 core eukaryotic genes, produced by the software CEGMA [7] with default parameters

Group	#Prots	%Completeness	#Total	Average	%Ortho
Complete	231	93.15	545	2.36	74.46
Group1	63	95.45	127	2.02	66.67
Group2	50	89.29	120	2.40	78.00
Group3	58	95.08	136	2.34	72.41
Group4	60	92.31	162	2.70	81.67
Partial	243	97.98	710	2.92	86.01
Group1	64	96.97	173	2.70	82.81
Group2	54	96.43	159	2.94	87.04
Group3	61	100.00	181	2.97	88.52
Group4	64	98.46	197	3.08	85.94

#Prots: number of 248 ultra-conserved CEGs present in genome

%Completeness: percentage of 248 ultra-conserved CEGs present

Total: total number of CEGs present including putative orthologs

Average: average number of orthologs per CEG

%Ortho: percentage of detected CEGS that have more than 1 ortholog

‘Complete’: predicted proteins in the set of 248 CEGs that, when aligned to the HMM (a hidden markov model) for the KOG (eukaryotic orthologous groups) for that protein family, give an alignment length that is at least 70 % of the protein length

‘Partial’: If a protein is not complete, but exceeds a pre-computed minimum alignment score, then we call the protein ‘partial’. The pre-computed scores are all in the file CEGMA/data/completeness_cutoff.tbl [7]

*CEG*s: core eukaryotic genes

## Repeat annotation of the ‘Golden Delicious’ apple genome assembly

Tandem Repeat Finder [8] was used to identify tandem repeats in the ‘Golden Delicious’ apple genome. RepeatMasker and RepeatProteinMasker [9] were used against Repbase [10] to identify known transposable element repeats. In addition, RepeatModeler [11] and LTR FINDER [12] were used to identify *de novo* evolved repeats. The combined results show that the total length of repeated sequences is about 382 Mb, accounting for ~60 % of the ‘Golden Delicious’ apple genome assembly (Additional file 1: Table S4).

## Gene annotation

Genes for the ‘Golden Delicious’ genome were annotated using multiple methods, including transcriptome-based predictions, *de novo* predictions, and homology-based predictions. For *de novo* predictions, Augustus [13], GenScan [14], glimmerHMM [15] and SNAP [16] analysis were performed on the repeat-masked genome, with parameters trained from *Arabidopsis thaliana*. Partial sequences and genes with fewer than 150 bp of coding sequence length were removed. Predicted protein sequences from *B. oleracea, G. max, O. sativa, P. mume, P. trichocarpa, P. persica, P. communis, V. vinifera,* and *Z. mays* were used (Phytozome v10.3 [17]) for homology-based predictions. First, query sequences were subjected to TBLASTN analysis with an Expect (E)-value cutoff of 1 e^-5^. BLAST hits corresponding to reference proteins were concatenated by Solar software (The Beijing Genomics Institute (BGI) development), and low-quality records were removed. The genomic sequence of each reference protein was extended upstream and downstream by 2,000 bp to represent a protein-coding region. GeneWise software [18] was used to predict gene structure contained in each protein region. For transcriptome-based predictions, RNA from three structures (leaves, flowers, and stems) was isolated and RNA-seq data (NCBI SRP067376) were used for gene annotation, processed by Tophat and Cufflinks [19]. The homology, *de novo* and transcriptomic gene sets were merged to form a comprehensive and non-redundant reference gene set using EVidenceModeler [20] software. Our analysis indicates that the ‘Golden Delicious’ apple genome contains 53,922 protein-coding genes (Table 2). This is slightly fewer than the previous prediction of 57,386 genes [6]. Approximately 60 % of predicted genes were represented in our transcriptome data.

**Table 2:** Statistics for ‘Golden Delicious’ genome protein-coding sequences annotation

		Gene_number	Avg_mRNA_length (bp)	Total_exon_number	Avg_exon_length (bp)	Avg_cds_length (bp)	Avg_exon_number	Total_intron_length (bp)
*De novo*	augustus	37693	2233.785106	203848	166.933235	902.793781	5.408113	50169056
	genscan	33206	8849.329489	210077	158.970511	1005.723303	6.326477	260454787
	glimmerHMM	48129	1404.407447	151751	182.492643	575.400299	3.153005	39899285
	snap	73555	936.269975	219634	162.207063	484.347577	2.985983	33241152
Homolog	*B. oleracea*	7000	2320.829429	46309	139.074802	920.059286	6.615571	9805391
	*G. max*	8578	2427.167172	60008	137.457522	961.593728	6.995570	12571689
	*O. sativa*	11000	1887.083182	61308	137.971668	768.978818	5.573455	12299148
	*P. mume*	9000	2623.029667	67760	135.473332	1019.963667	7.528889	14427594
	*P. trichocarpa*	30585	2321.131764	207830	138.869210	943.638646	6.795161	42130627
	*P. persica*	12733	2431.885573	93666	134.420665	988.820074	7.356161	18374553
	*P. communis*	34642	2833.118267	256347	129.467222	958.043242	7.399890	64956349
	*V. vinifera*	17175	2460.852402	118296	138.772773	955.823231	6.887686	25848876
	*Z. mays*	22341	2004.558569	130795	138.548645	811.130657	5.854483	26662373
RNA-seq	GDflorwer1	48423	2234.847387	212811	300.027231	1318.569585	4.394833	49998557
	GDflorwer2	49952	2231.126001	220057	304.286867	1340.495976	4.405369	50837822
	GDflorwer3	49848	2242.481785	223056	305.307031	1366.164440	4.474723	49515976
	GDleaf1	45034	2258.958920	203894	296.653634	1343.116223	4.527557	46765622
	GDleaf2	44669	2300.217086	204106	298.250576	1362.795943	4.569299	47700782
	GDleaf3	45220	2292.436975	206566	301.208723	1375.928372	4.568023	47304519
	GDstem1	46908	2299.298840	212019	308.944807	1396.396542	4.519890	48015182
	GDstem2	46271	2308.347604	209286	307.787090	1392.136090	4.523049	48368862
	GDstem3	46657	2296.511542	209284	310.624348	1393.332319	4.485586	48454706
EVM		53922	1793.161066	221394	167.775983	688.857906	4.105820	59546235

## Non-coding RNA annotation

tRNAscan-SE (version 1.31) [21] software with default parameters for eukaryotes was used for tRNA annotation. rRNA annotation was based on homology with rRNAs from several diverse higher plant species (not shown), using BLASTN with ‘E-value = 1e^-5^’. miRNA and snRNA genes were predicted by INFERNAL software [22] using the Rfam database (release 11.0) [23]. The final results included 321 miRNAs, 274 tRNAs, 605 rRNAs, and 480 snRNAs (Additional file 1: Table S5).

## Availability of supporting data

Sequencing reads of each sequencing library and RNA-seq data have been deposited at NCBI with the project ID SRP067376. Supporting data are also available in the GigaScience database, GigaDB [24]. All supplementary figures and tables are provided in Additional file 1.

## Abbreviations

CDS, coding DNA sequence; NCBI, National Center for Biotechnology Information

## Additional file

Additional file 1: Supplementary figures and tables. (ZIP 326 kb)
