# Supplementary material for: Improved hybrid de novo genome assembly of domesticated apple (Malus x domestica)
Source: Gigascience. 2016 Aug 8;5:35. doi: 10.1186/s13742-016-0139-0 (PMC4976516; doi:10.1186/s13742-016-0139-0)
Supplement: Additional file 1: — Supplementary figures and tables. (ZIP 326 kb) [file 13742_2016_139_MOESM1_ESM.zip › Supplementary Table 1R2.pdf]

| | Insert_ size | Raw_reads | Raw_bases | Clean_Reads | Clean_Bases | Corrected_Reads | Corrected_Bases | Coverage | Contig N50 （bp） | Genome size (Mb) | Estimated genome size (Mb) |
|---|---|---|---|---|---|---|---|---|---|---|---|
| Illumina | 350 bp | 301,001,150 | 45,150,172,500 | 279,108,332 | 41,866,249,800 | 276,663,486 | 40,534,047,978 | 102 | 534 | 632.4 | 701 |
| | 350 bp | 273,532,258 | 41,029,838,700 | 253,324,488 | 37,998,673,200 | 251,042,920 | 36,784,658,014 | | | | |
| PacBio | 17 Kb | 2,759,937 | 21,701,412,596 | * | * | * | * | 29 | 111,619 | | |

Coverage was calculated on the basis of a genome size of 742.3 Mb.

*, not available.
