# Supplementary material for: Improved hybrid de novo genome assembly of domesticated apple (Malus x domestica)
Source: Gigascience. 2016 Aug 8;5:35. doi: 10.1186/s13742-016-0139-0 (PMC4976516; doi:10.1186/s13742-016-0139-0)
Supplement: Additional file 1: — Supplementary figures and tables. (ZIP 326 kb) [file 13742_2016_139_MOESM1_ESM.zip › Supplementary Table 2R2.pdf]

Assembly versions	Total_length (bp)	Cotig N50 (bp)	Sequences
DBG2OLC_raw	666334801	117136	9675
DBG2OLC_consensus	632422011	111619	9675
Malus_x_domestica.v1.0	881278625	11136	122107
