# Supplementary material for: Improved hybrid de novo genome assembly of domesticated apple (Malus x domestica)
Source: Gigascience. 2016 Aug 8;5:35. doi: 10.1186/s13742-016-0139-0 (PMC4976516; doi:10.1186/s13742-016-0139-0)
Supplement: Additional file 1: — Supplementary figures and tables. (ZIP 326 kb) [file 13742_2016_139_MOESM1_ESM.zip › Supplementary Table 3R2.pdf]

Group	#Prots	%Completeness	#Total	Average	%Ortho
Complete	220	88.71	696	3.16	84.55
Group1	58	87.88	171	2.95	84.48
Group2	52	92.86	173	3.33	82.69
Group3	55	90.16	158	2.87	81.82
Group4	55	84.62	194	3.53	89.09
Partial	239	96.37	920	3.85	94.98
Group1	64	96.97	240	3.75	95.31
Group2	56	100.00	219	3.91	91.07
Group3	59	96.72	225	3.81	96.61
Group4	60	92.31	236	3.93	96.67

#Prots: number of 248 ultra-conserved CEGs present in genome.

%Completeness: percentage of 248 ultra-conserved CEGs present.

Total: total number of CEGs present including putative orthologs.

Average: average number of orthologs per CEG.

%Ortho: percentage of detected CEGS that have more than 1 ortholog.

'Complete': predicted proteins in the set of 248 CEGs that, when aligned to the HMM (hidden markov model) for the KOG (eukaryotic orthologous groups) for that protein family, give an alignment length that is at least 70% of the protein length.

'Partial': If a protein is not complete, but exceeds a pre-computed minimum alignment score, then we call the protein 'partial'. The pre-computed scores are all in the file CEGMA/data/completeness_cutoff.tbl [7].
