# Supplementary material for: Improved hybrid de novo genome assembly of domesticated apple (Malus x domestica)
Source: Gigascience. 2016 Aug 8;5:35. doi: 10.1186/s13742-016-0139-0 (PMC4976516; doi:10.1186/s13742-016-0139-0)
Supplement: Additional file 1: — Supplementary figures and tables. (ZIP 326 kb) [file 13742_2016_139_MOESM1_ESM.zip › Supplementary Table 4R2.pdf]

	Repbase TEs		TE protiens		*De novo*		Combined TEs	
	Length (bp)	% in genome	Length (bp)	% in genome	Length (bp)	% in genome	Length (bp)	% in genome
DNA	3913723	0.618847	7255342	1.147231	74230930	11.737563	77785611	12.299637
LINE	822252	0.130016	10387318	1.642466	27297328	4.316315	29759036	4.705566
SINE	21172	0.003348	0	0	470742	0.074435	488999	0.077322
LTR	42726151	6.755956	66995832	10.593533	290852356	45.990233	292483794	46.248200
Other	1028	0.000163	0	0	0	0	1028	0.000163
Unknown	0	0	0	0	13992572	2.212537	13992572	2.212537
Total	47298803	7.478994	84487776	13.359398	379356464	59.984703	382472795	60.477464

DNA, DNA transposons; LINE, long interspersed nuclear elements; TEs, transposable elements;
SINE, short interspersed nuclear elements; LTR, long terminal repeats.
