# Supplementary material for: Improved hybrid de novo genome assembly of domesticated apple (Malus x domestica)
Source: Gigascience. 2016 Aug 8;5:35. doi: 10.1186/s13742-016-0139-0 (PMC4976516; doi:10.1186/s13742-016-0139-0)
Supplement: Additional file 1: — Supplementary figures and tables. (ZIP 326 kb) [file 13742_2016_139_MOESM1_ESM.zip › Supplementary Table 5R2.pdf]

	miRNA	tRNA	rRNA				snRNA			Total
			18S	28S	5.8S	5S	CD-box	HACA-box	splicing	
Copy (w)	321	274	114	87	35	369	281	47	152	1680
Average length (bp)	124.984423	75.182481	429.745614	109.206896	111.371428	95.813008	100.508897	114.893617	150.493421	1312.199785
Total length (bp)	40120	20600	48991	9501	3898	35355	28243	5400	22875	214983
% of genome	0.006344	0.003257	0.007747	0.001502	0.000616	0.005590	0.004466	0.000854	0.003617	0.033993

miRNA, microRNA; tRNA, transfer RNA; rRNA, ribosomal RNA; snRNA, small nuclear RNA.
