# Supplementary figures and images for: Improved hybrid de novo genome assembly of domesticated apple (Malus x domestica)
Source: Gigascience. 2016 Aug 8;5:35. doi: 10.1186/s13742-016-0139-0 (PMC4976516; doi:10.1186/s13742-016-0139-0)

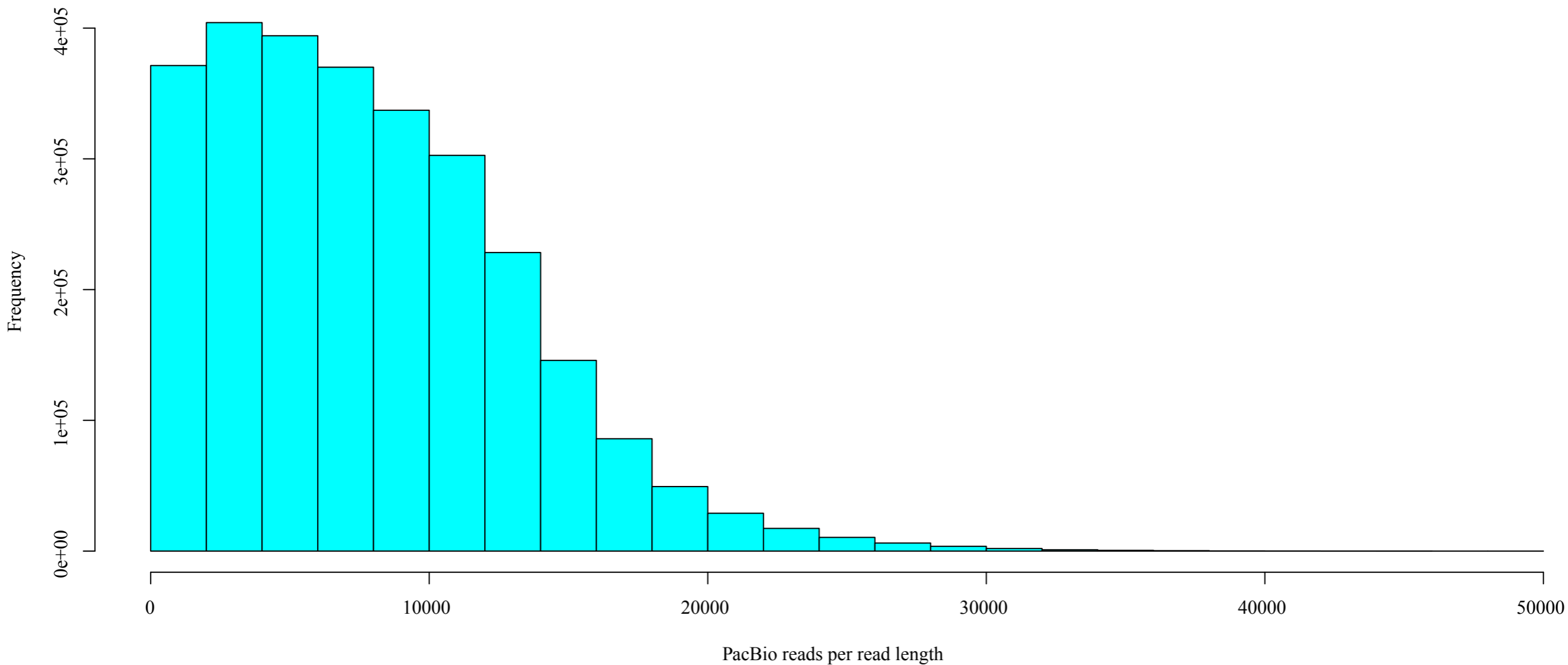

Supplement: Additional file 1: — Supplementary figures and tables. (ZIP 326 kb) [file 13742_2016_139_MOESM1_ESM.zip › Supplementary Figure 1R2.pdf]

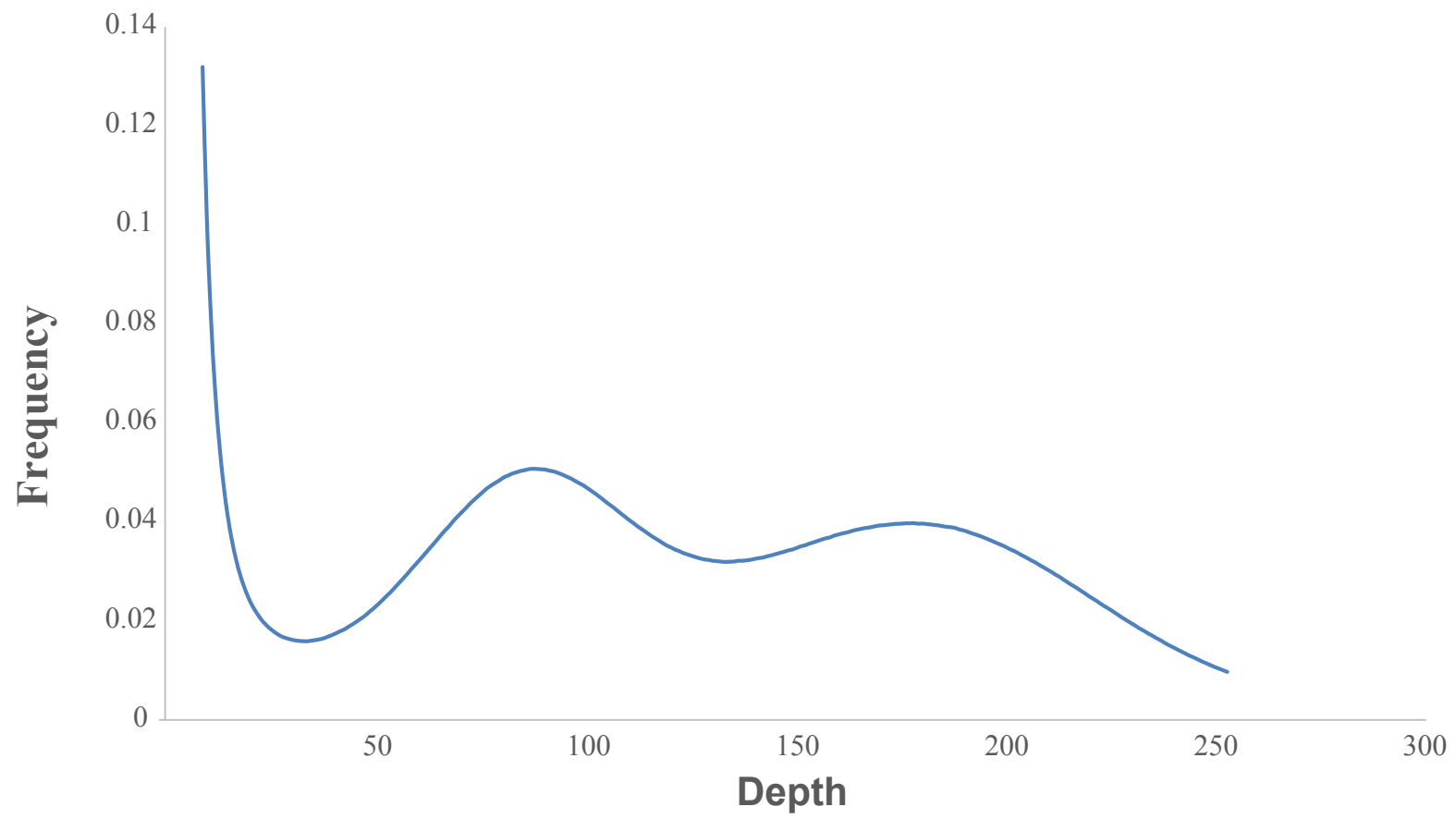

Supplement: Additional file 1: — Supplementary figures and tables. (ZIP 326 kb) [file 13742_2016_139_MOESM1_ESM.zip › Supplementary Figure 2R2.pdf]

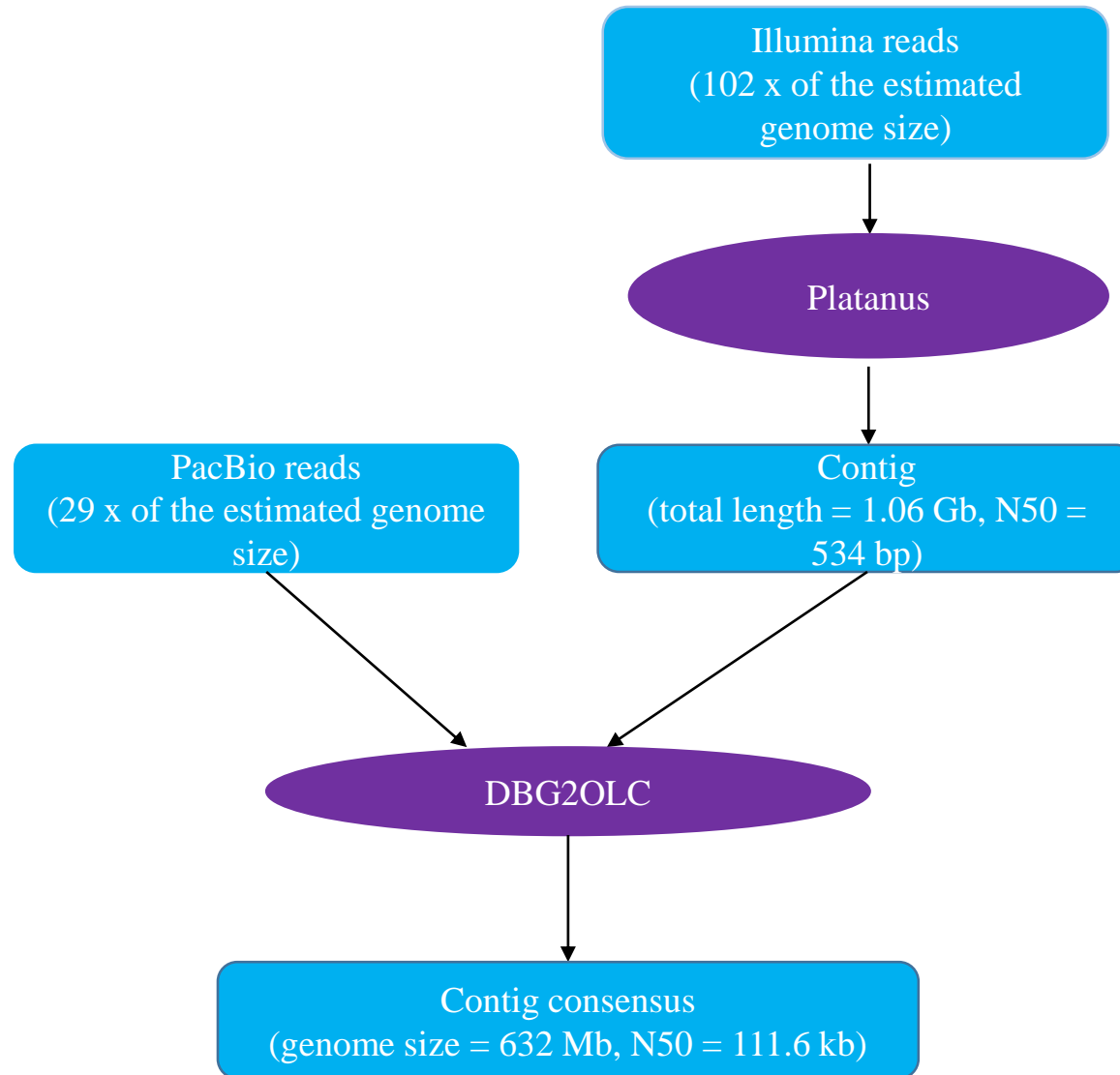

Supplement: Additional file 1: — Supplementary figures and tables. (ZIP 326 kb) [file 13742_2016_139_MOESM1_ESM.zip › Supplementary Figure 3R2.pdf]

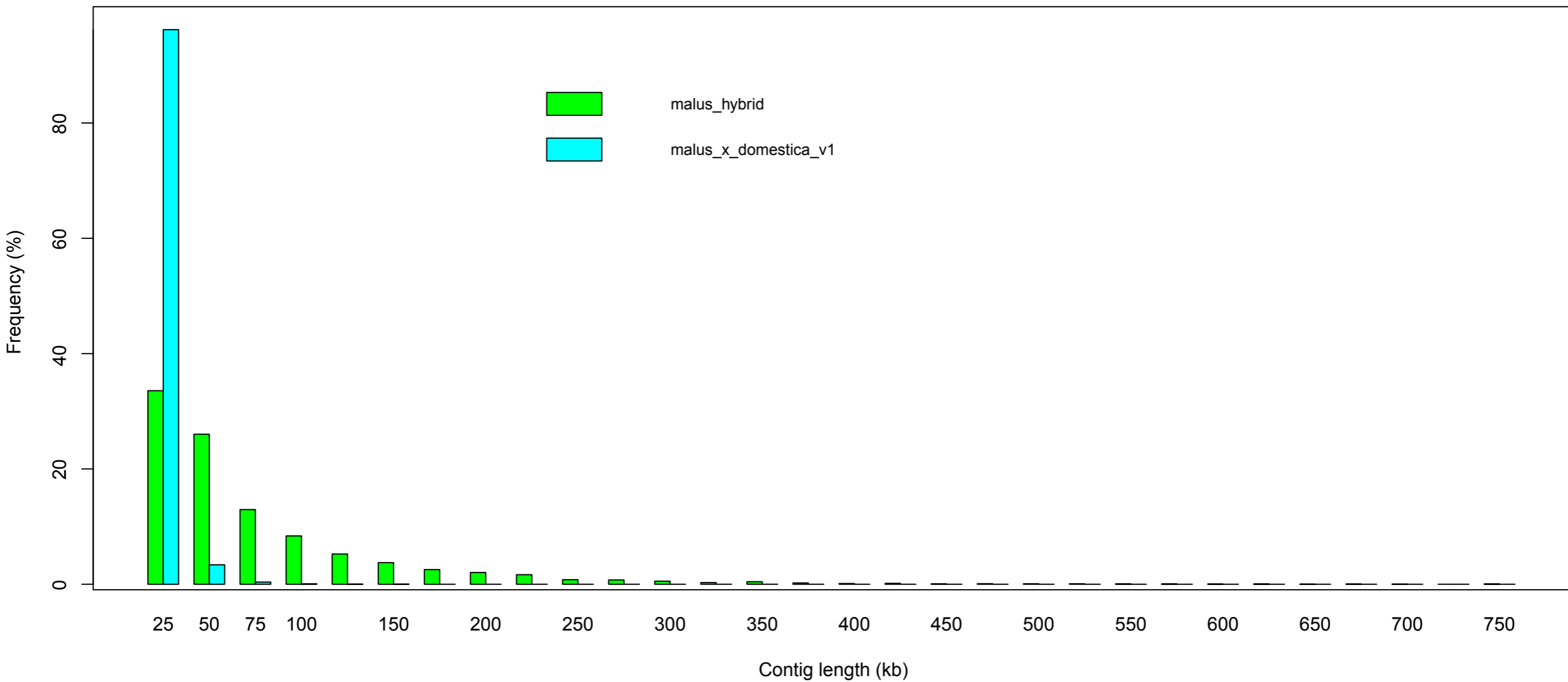

Supplement: Additional file 1: — Supplementary figures and tables. (ZIP 326 kb) [file 13742_2016_139_MOESM1_ESM.zip › Supplementary Figure 4R2.pdf]
